# Supplementary material for: A Likelihood Approach for Real-Time Calibration of Stochastic Compartmental Epidemic Models
Source: PLoS Comput Biol. 2017 Jan 17;13(1):e1005257. doi: 10.1371/journal.pcbi.1005257 (PMC5240920; doi:10.1371/journal.pcbi.1005257)
Supplement: S1 File — (TAR.GZ) [file pcbi.1005257.s014.tar.gz › HSPH_Online-SI-Revision/IPoi09/n100-mild/n100-mild_BENCH_table4.pdf]

1	R0	{1.24125, 3.38982}	{1.85533, 9.28761}	{{1.85533, 9.28761}, {1.85533, 9.28761}}	{49.4727, 173.985}	inf	918.	5616.5	{2455., 10017.}	511.819	0.736078	newly	1628.	3676.21	{1505., 6743.}	129.597	0.313768	Reff	0.854936	0.586055	{0.497952, 0.7192}	33.0107	ar	34926.	75291.3	{74879., 75728.}	115.574
2	R0	{1.29554, 4.51878}	{2.12134, 12.1725}	{{2.12117, 12.1677}, {2.12117, 12.1677}}	{63.7415, 169.377}	inf	1374.	7965.24	{3646., 13339.}	479.711	0.715311	newly	1875.	4087.8	{1754., 6999.}	121.003	0.295626	Reff	0.799646	0.534686	{0.416296, 0.652281}	38.7267	ar	42828.	82653.7	{82384., 82924.}	92.9898
3	R0	{1.2443, 3.64292}	{1.93419, 8.56054}	{{1.93419, 8.56054}, {1.93419, 8.56054}}	{55.444, 134.991}	inf	748.	3897.66	{1929., 6066.}	421.077	0.674405	newly	1275.	2614.12	{1239., 4060.}	108.606	0.284562	Reff	0.825668	0.494775	{0.458925, 0.53695}	40.0758	ar	37087.	77863.7	{77421., 78275.}	109.949
4	R0	{1.38465, 3.43331}	{2.20704, 7.29612}	{{2.20704, 7.29612}, {2.20704, 7.29612}}	{59.3933, 112.51}	inf	741.	2554.05	{1006., 5236.}	244.745	0.453143	newly	1149.	1853.47	{720., 3818.}	80.1361	0.223437	Reff	0.722441	0.373853	{0.348668, 0.411392}	48.2514	ar	49511.	84560.8	{84251., 84856.}	70.7919
5	R0	{1.25409, 4.1068}	{1.93419, 8.56054}	{{1.93419, 8.56054}, {1.93419, 8.56054}}	{54.2306, 108.448}	inf	1115.	3897.66	{1929., 6066.}	249.566	0.501032	newly	1641.	2614.12	{1239., 4060.}	69.6185	0.211421	Reff	0.822708	0.494775	{0.458925, 0.53695}	39.8602	ar	38571.	77863.7	{77421., 78275.}	101.871
6	R0	{1.28821, 4.2016}	{2.01028, 10.1103}	{{2.01028, 10.1103}, {2.01028, 10.1103}}	{56.0522, 140.63}	inf	1013.	5341.96	{2454., 9883.}	427.34	0.663586	newly	1399.	3163.31	{1400., 5961.}	128.572	0.301305	Reff	0.800326	0.497863	{0.433396, 0.598521}	38.0988	ar	41468.	80025.5	{79657., 80441.}	92.9812
7	R0	{1.24369, 2.56007}	{2.13505, 8.31782}	{{2.13505, 8.31782}, {2.13505, 8.31782}}	{71.6706, 224.906}	inf	442.	4984.88	{2342., 9291.}	1027.8	0.993354	newly	924.	3359.48	{1537., 6349.}	263.58	0.496893	Reff	0.781634	0.436329	{0.390074, 0.511771}	44.1773	ar	38603.	83073.6	{82770., 83368.}	115.2
8	R0	{1.232, 3.53942}	{1.93816, 10.0503}	{{1.93816, 10.0503}, {1.93816, 10.0503}}	{57.3182, 183.953}	inf	649.	4728.82	{2245., 8747.}	628.632	0.804685	newly	1057.	2850.73	{1296., 5332.}	170.886	0.37036	Reff	0.831846	0.534139	{0.460371, 0.593406}	39.1164	ar	34555.	77897.5	{77498., 78370.}	125.431
9	R0	{1.36262, 3.85631}	{2.33832, 9.79994}	{{2.33832, 9.79994}, {2.33832, 9.79994}}	{71.6047, 154.127}	inf	951.	6134.91	{2724., 12848.}	545.101	0.731495	newly	1368.	3662.25	{1520., 7567.}	170.383	0.350213	Reff	0.74132	0.408041	{0.333211, 0.5004}	49.226	ar	48410.	86902.8	{86650., 87184.}	79.5141
10	R0	{1.28702, 4.67968}	{1.98131, 11.1772}	{{1.98131, 11.1772}, {1.98131, 11.1772}}	{53.9455, 138.845}	inf	1183.	5231.28	{2200., 10214.}	342.28	0.564963	newly	1557.	2860.19	{1145., 5605.}	96.3063	0.249372	Reff	0.80682	0.511621	{0.441852, 0.627283}	36.588	ar	41092.	79152.9	{78853., 79488.}	92.6236
11	R0	{1.35752, 3.47129}	{2.27199, 11.6669}	{{2.27199, 11.6669}, {2.27199, 11.6669}}	{67.3633, 236.097}	inf	1161.	9161.64	{4780., 14965.}	689.116	0.860864	newly	1963.	4802.78	{2402., 7850.}	145.48	0.354183	Reff	0.799932	0.524738	{0.3794, 0.643927}	45.3928	ar	44669.	85794.3	{85446., 86067.}	92.0667
12	R0	{1.35929, 5.83647}	{2.18932, 13.5121}	{{2.18932, 13.5121}, {2.18932, 13.5121}}	{61.0635, 131.512}	inf	1940.	9571.81	{4563., 16656.}	393.393	0.647186	newly	2036.	4470.43	{2034., 7998.}	121.856	0.298739	Reff	0.813535	0.5346	{0.408921, 0.735852}	37.4866	ar	46715.	84192.2	{83771., 84524.}	80.2252
13	R0	{1.28019, 3.29908}	{2.06386, 8.81239}	{{2.06386, 8.81239}, {2.06386, 8.81239}}	{61.2151, 167.117}	inf	878.	4447.4	{2188., 7227.}	406.538	0.648522	newly	1478.	2873.06	{1397., 4724.}	100.269	0.262235	Reff	0.791618	0.454452	{0.40852, 0.499846}	42.592	ar	40941.	81347.9	{81053., 81619.}	98.6955
14	R0	{1.36661, 3.48318}	{2.26582, 8.12562}	{{2.26582, 8.12562}, {2.26582, 8.12562}}	{65.7986, 133.282}	inf	608.	3256.88	{917., 6060.}	435.67	0.614436	newly	929.	2216.41	{596., 4080.}	149.356	0.308706	Reff	0.726053	0.373182	{0.337517, 0.408301}	49.7291	ar	48672.	85637.7	{85350., 86000.}	75.9487
15	R0	{1.35865, 3.84123}	{2.43464, 12.8735}	{{2.43464, 12.8735}, {2.43464, 12.8735}}	{79.1955, 235.14}	inf	1150.	11224.	{5725., 20678.}	875.999	0.95001	newly	1651.	5458.44	{2625., 10748.}	230.614	0.471031	Reff	0.73825	0.520369	{0.343163, 0.819865}	45.4749	ar	48598.	88395.5	{88143., 88603.}	81.8911
16	R0	{1.22879, 3.98329}	{1.77826, 9.06807}	{{1.77826, 9.06807}, {1.77826, 9.06807}}	{44.7163, 127.653}	inf	1035.	3840.8	{1719., 6592.}	271.092	0.51008	newly	1786.	2555.73	{1097., 4399.}	61.53	0.190426	Reff	0.876398	0.575286	{0.51614, 0.652248}	34.3579	ar	34405.	72400.8	{71885., 72704.}	110.437
17	R0	{1.34925, 3.64524}	{2.22376, 9.35432}	{{2.22376, 9.35432}, {2.22376, 9.35432}}	{64.8145, 156.617}	inf	1239.	6078.25	{3041., 10575.}	390.577	0.625639	newly	1851.	3776.32	{1793., 6651.}	108.072	0.255594	Reff	0.759911	0.435287	{0.371168, 0.500124}	44.662	ar	46871.	84873.7	{84562., 85179.}	81.0794
18	R0	{1.33054, 2.25275}	{2.17292, 6.16453}	{{2.17292, 6.16453}, {2.17292, 6.16453}}	{63.3111, 173.645}	inf	312.	1924.04	{861., 3271.}	516.679	0.725561	newly	665.	1571.	{717., 2691.}	138.028	0.316737	Reff	0.750025	0.375893	{0.357684, 0.393451}	49.8826	ar	44336.	83825.5	{83511., 84088.}	89.0688
19	R0	{1.28394, 3.69859}	{1.98131, 11.1772}	{{1.98131, 11.1772}, {1.98131, 11.1772}}	{54.3148, 202.202}	inf	963.	8395.11	{4421., 14490.}	771.766	0.894	newly	1523.	4737.64	{2367., 8628.}	212.073	0.442138	Reff	0.824444	0.616913	{0.481399, 0.856917}	29.8519	ar	39558.	79152.9	{78853., 79488.}	100.093
20	R0	{1.2883, 2.26493}	{1.98552, 5.56151}	{{1.98552, 5.56151}, {1.98552, 5.56151}}	{54.1194, 145.549}	inf	531.	3179.06	{984., 8268.}	498.902	0.641906	newly	1246.	2991.87	{863., 7970.}	154.721	0.309719	Reff	0.78634	0.47013	{0.421268, 0.571016}	40.3331	ar	40768.	79224.6	{78759., 79585.}	94.3302
21	R0	{1.34098, 2.78621}	{2.17292, 6.16453}	{{2.17292, 6.16453}, {2.17292, 6.16453}}	{62.0397, 121.251}	inf	549.	1924.04	{861., 3271.}	250.462	0.480143	newly	1041.	1571.	{717., 2691.}	67.5367	0.202406	Reff	0.763192	0.375893	{0.357684, 0.393451}	50.7472	ar	44605.	83825.5	{83511., 84088.}	87.9285
22	R0	{1.33783, 5.95911}	{2.11132, 12.644}	{{2.11132, 12.644}, {2.11132, 12.644}}	{57.8168, 112.179}	inf	1990.	8314.52	{4286., 14583.}	317.815	0.578396	newly	2166.	4081.81	{2041., 7371.}	92.1053	0.244719	Reff	0.826485	0.524948	{0.423953, 0.704949}	37.1643	ar	45418.	82482.7	{82204., 82804.}	81.608
23	R0	{1.33359, 3.3069}	{2.34121, 11.1899}	{{2.34121, 11.1899}, {2.34121, 11.1899}}	{75.557, 238.38}	inf	1009.	8960.73	{4176., 17044.}	788.08	0.890143	newly	1653.	4852.02	{2207., 9581.}	194.527	0.405141	Reff	0.751291	0.46774	{0.353546, 0.651371}	42.9725	ar	46245.	86920.	{86648., 87194.}	87.9555
24	R0	{1.30588, 2.56123}	{2.0598, 5.9936}	{{2.0598, 5.9936}, {2.0598, 5.9936}}	{57.7327, 134.013}	inf	372.	1661.16	{579., 3224.}	346.548	0.554807	newly	702.	1401.34	{482., 2734.}	112.954	0.273528	Reff	0.762347	0.408909	{0.390476, 0.429077}	46.3619	ar	42383.	81278.	{80868., 81557.}	91.7702
25	R0	{1.29813, 4.51849}	{2.22641, 14.8348}	{{2.22641, 14.8348}, {2.22641, 14.8348}}	{71.509, 228.313}	inf	1528.	13302.8	{7537., 18370.}	770.6	0.912594	newly	2037.	5823.33	{3077., 8379.}	187.28	0.435192	Reff	0.813343	0.683843	{0.454121, 0.968199}	32.2922	ar	43371.	84916.4	{84644., 85184.}	95.7908
26	R0	{1.33231, 3.99953}	{2.18735, 12.1087}	{{2.18735, 12.1087}, {2.18735, 12.1087}}	{64.1773, 202.753}	inf	1265.	8636.81	{4930., 14739.}	582.752	0.789389	newly	1843.	4405.43	{2408., 7598.}	140.362	0.331648	Reff	0.789434	0.510894	{0.40711, 0.638816}	38.699	ar	44733.	84157.2	{83791., 84464.}	88.1323
27	R0	{1.30555, 4.61445}	{2.30088, 15.2916}	{{2.21411, 14.2212}, {2.315, 15.4658}}	{76.238, 231.384}	inf	1370.	13027.5	{6461., 19421.}	850.91	0.945451	newly	1833.	5475.43	{2504., 8840.}	198.878	0.441193	Reff	0.814154	0.624614	{0.404616, 1.04055}	37.0342	ar	42624.	86189.9	{84668., 86731.}	102.21
28	R0	{1.26819, 5.34969}	{1.87927, 9.84456}	{{1.87927, 9.84456}, {1.87927, 9.84456}}	{48.1852, 84.0211}	inf	1478.	5651.81	{2727., 10963.}	282.755	0.522186	newly	1792.	3535.3	{1619., 7074.}	104.127	0.260791	Reff	0.858552	0.586817	{0.492575, 0.739793}	34.4277	ar	38946.	75982.9	{75584., 76490.}	95.098
29	R0	{1.19925, 3.00394}	{1.75445, 7.55154}	{{1.75445, 7.55154}, {1.75445, 7.55154}}	{46.2956, 151.388}	inf	570.	3164.73	{1414., 6297.}	455.216	0.667899	newly	1093.	2471.88	{1052., 4931.}	130.096	0.288103	Reff	0.866182	0.573407	{0.522844, 0.638392}	34.6571	ar	31727.	71490.9	{70900., 71883.}	125.332
30	R0	{1.35749, 3.75483}	{2.52289, 12.2023}	{{2.52588, 12.2207}, {2.52588, 12.2207}}	{85.8494, 224.976}	inf	1015.	8945.66	{4936., 14049.}	781.346	0.898012	newly	1413.	4448.79	{2382., 6873.}	214.847	0.43988	Reff	0.732529	0.412036	{0.31278, 0.452511}	51.7774	ar	48244.	89532.8	{89282., 89736.}	85.5832
31	R0	{1.27131, 4.2161}	{2.0072, 10.6431}	{{2.0072, 10.6431}, {2.0072, 10.6431}}	{57.8844, 152.439}	inf	1215.	6879.19	{3691., 11338.}	466.189	0.719486	newly	1803.	3934.15	{2029., 6667.}	120.584	0.311593	Reff	0.843883	0.551935	{0.455514, 0.65513}	38.4168	ar	38299.	79852.6	{79507., 80196.}	108.498
32	R0	{1.31827, 6.18119}	{1.98131, 11.1772}	{{1.98131, 11.1772}, {1.98131, 11.1772}}	{50.2962, 80.826}	inf	1873.	6750.21	{3161., 12725.}	260.923	0.494479	newly	1902.	3740.49	{1702., 7281.}	104.373	0.265082	Reff	0.818804	0.555636	{0.458931, 0.721791}	33.4718	ar	45262.	79152.9	{78853., 79488.}	74.8771
33	R0	{1.20398, 1.35167}	{1.82289, 4.40955}	{{1.82289, 4.40955}, {1.82289, 4.40955}}	{51.4053, 226.23}	inf	90.	819.756	{294., 1602.}	810.84	0.85121	newly	298.	894.111	{317., 1750.}	202.975	0.380426	Reff	0.806895	0.486808	{0.468701, 0.502644}	39.6689	ar	33329.	74065.1	{73640., 74632.}	122.224
34	R0	{1.32776, 3.146}	{2.25325, 9.0684}	{{2.25325, 9.0684}, {2.25325, 9.0684}}	{69.7031, 188.252}	inf	466.	4804.75	{1806., 9204.}	931.062	0.921219	newly	799.	3021.04	{1075., 5891.}	278.911	0.480651	Reff	0.745949	0.399882	{0.346798, 0.463741}	46.9186	ar	44872.	85381.9	{85123., 85664.}	90.2788
35	R0	{1.279, 1.11336}	{2.00921, 5.05524}	{{2.00921, 5.05524}, {2.00921, 5.05524}}	{57.0923, 354.053}	inf	15.	1012.96	{321., 1726.}	6653.06	1.70373	newly	47.	968.531	{292., 1657.}	1960.7	1.18466	Reff	0.754597	0.417766	{0.401521, 0.430292}	44.6372	ar	41033.	79930.1	{79603., 80309.}	94.7947
36	R0	{1.20029, 1.94717}	{1.79887, 4.61852}	{{1.79887, 4.61852}, {1.79887, 4.61852}}	{49.8696, 137.191}	inf	338.	1346.16	{574., 3056.}	298.273	0.513754	newly	910.	1441.	{626., 3332.}	78.0041	0.216271	Reff	0.82688	0.506964	{0.486019, 0.539355}	38.6895	ar	33234.	73152.6	{72671., 73553.}	120.114
37	R0	{1.33216, 3.69685}	{2.2885, 12.1239}	{{2.2885, 12.1239}, {2.2885, 12.1239}}	{71.7887, 227.952}	inf	945.	8963.58	{4178., 14784.}	848.527	0.930587	newly	1507.	4524.24	{2073., 7780.}	200.524	0.42672	Reff	0.770335	0.481921	{0.370691, 0.608855}	42.2537	ar	45235.	86072.9	{85775., 86371.}	90.2794
38	R0	{1.25434, 2.04314}	{2.02938, 5.7296}	{{2.02938, 5.7296}, {2.02938, 5.7296}}	{61.7887, 180.431}	inf	246.	2614.57	{1064., 4941.}	962.834	0.94797	newly	612.	2318.27	{920., 4417.}	279.655	0.498552	Reff	0.777829	0.43677	{0.41032, 0.476295}	43.8476	ar	38662.	80417.2	{80074., 80858.}	108.001
39	R0	{1.27466, 3.0741}	{1.89934, 6.19362}	{{1.89934, 6.19362}, {1.89934, 6.19362}}	{49.0076, 101.478}	inf	499.	1758.31	{606., 3257.}	252.672	0.446776	newly	937.	1509.33	{524., 2886.}	88.5348	0.254049	Reff	0.783495	0.472127	{0.450751, 0.506079}	39.7409	ar	40181.	76667.4	{76233., 77080.}	90.8051
40	R0	{1.33045, 2.68154}	{2.27423, 6.876}	{{2.27423, 6.876}, {2.27423, 6.876}}	{70.9369, 156.42}	inf	318.	2366.65	{793., 4602.}	644.229	0.732321	newly	616.	1819.94	{591., 3452.}	198.53	0.332811	Reff	0.729978	0.35608	{0.329422, 0.37618}	51.6092	ar	45951.	85776.6	{85543., 86036.}	86.6697
41	R0	{1.37003, 2.83984}	{2.33614, 6.89476}	{{2.33614, 6.89476}, {2.33614, 6.89476}}	{70.5174, 142.787}	inf	516.	3279.56	{1163., 6523.}	535.573	0.698451	newly	894.	2479.76	{819., 4872.}	180.721	0.344991	Reff	0.718704	0.348625	{0.315916, 0.390486}	51.4926	ar	48926.	86878.2	{86567., 87224.}	77.5707
42	R0	{1.3003, 2.94885}	{2.19024, 8.25876}	{{2.19024, 8.25876}, {2.19024, 8.25876}}	{68.4411, 180.067}	inf	576.	3994.24	{1620., 6728.}	593.445	0.776032	newly	1090.	2668.07	{1109., 4510.}	147.064	0.330649	Reff	0.746177	0.40102	{0.366865, 0.444159}	46.2568	ar	44237.	84214.3	{83888., 84492.}	90.3708
43	R0	{1.33756, 2.92653}	{2.27423, 6.876}	{{2.27423, 6.876}, {2.27423, 6.876}}	{70.0283, 134.954}	inf	776.	4126.41	{1548., 8555.}	431.754	0.62214	newly	1372.	3230.17	{1138., 6627.}	143.417	0.291028	Reff	0.730161	0.38952	{0.338587, 0.43713}	49.3615	ar	47342.	85776.6	{85543., 86036.}	81.185
44	R0	{1.35801, 2.92276}	{2.25976, 7.03829}	{{2.25976, 7.03829}, {2.25976, 7.03829}}	{66.4023, 140.81}	inf	436.	3014.05	{1012., 5322.}	591.297	0.725302	newly	782.	2272.91	{729., 3973.}	194.773	0.358284	Reff	0.722081	0.368655	{0.335077, 0.395955}	49.3117	ar	47940.	85531.9	{85262., 85845.}	78.4145
45	R0	{1.31342, 4.02649}	{2.03307, 8.59564}	{{2.03307, 8.59564}, {2.03307, 8.59564}}	{54.7921, 113.477}	inf	1233.	4714.54	{1807., 8341.}	282.363	0.513352	newly	1772.	3157.21	{1178., 5635.}	91.7419	0.247083	Reff	0.7963	0.477904	{0.42174, 0.548685}	40.1938	ar	42995.	80504.1	{80124., 80869.}	87.2407
46	R0	{1.37919, 3.75889}	{2.45095, 11.4067}	{{2.45095, 11.4067}, {2.45095, 11.4067}}	{77.7094, 203.459}	inf	1312.	10431.8	{4980., 18510.}	695.103	0.859018	newly	1998.	5554.85	{2540., 10081.}	178.021	0.393884	Reff	0.747438	0.469528	{0.33627, 0.62808}	46.3231	ar	49157.	88594.2	{88354., 88823.}	80.2271
47	R0	{1.37458, 3.56117}	{2.22376, 9.35432}	{{2.22376, 9.35432}, {2.22376, 9.35432}}	{61.7774, 162.675}	inf	838.	6078.25	{3041., 10575.}	625.328	0.795467	newly	1272.	3776.32	{1793., 6651.}	196.908	0.39845	Reff	0.732981	0.435287	{0.371168, 0.500124}	42.7577	ar	48478.	84873.7	{84562., 85179.}	75.0768
48	R0	{1.33393, 3.78296}	{2.46246, 15.904}	{{2.46246, 15.904}, {2.46246, 15.904}}	{84.6019, 320.412}	inf	1093.	14573.3	{8778., 21807.}	1233.33	1.10177	newly	1686.	5884.33	{3383., 9076.}	249.011	0.514406	Reff	0.779095	0.57136	{0.382125, 0.901383}	38.1447	ar	44885.	88733.5	{88507., 88940.}	97.6907
49	R0	{1.24918, 2.39046}	{1.84836, 6.13642}	{{1.84836, 6.13642}, {1.84836, 6.13642}}	{47.9659, 156.705}	inf	342.	2176.26	{860., 3585.}	536.332	0.694729	newly	806.	1937.79	{730., 3185.}	145.684	0.286534	Reff	0.817189	0.50484	{0.472071, 0.530775}	38.4386	ar	35969.	74960.1	{74591., 75406.}	108.402
50	R0	{1.28281, 2.3032}	{1.98388, 6.25659}	{{1.98388, 6.25659}, {1.98388, 6.25659}}	{54.6511, 171.648}	inf	333.	2391.98	{753., 6151.}	618.311	0.706233	newly	744.	2025.86	{594., 5297.}	180.951	0.322426	Reff	0.782989	0.451393	{0.417408, 0.522316}	42.35	ar	40223.	79238.6	{78771., 79670.}	96.9981
